# Nanosized Pinning Centers in the Rare Earth-Barium-Copper-Oxide Thin-Film Superconductors

**DOI:** 10.3390/nano10081429

**Published:** 2020-07-22

**Authors:** Filip Antončík, Ondřej Jankovský, Tomáš Hlásek, Vilém Bartůněk

**Affiliations:** 1 Department of Inorganic Chemistry, University of Chemistry and Technology, Technická 5, 166 28 Prague 6, Czech Republic; filip.antoncik@vscht.cz (F.A.); ondrej.jankovsky@vscht.cz (O.J.); tomas.hlasek@can-superconductors.com (T.H.); 2 CAN SUPERCONDUCTORS s.r.o., Ringhofferova 66, 251 68 Kamenice, Czech Republic

**Keywords:** HTS superconductors, thin-films, pinning, nano-objects, nanoparticles, YBCO, cuprates, REBCO

## Abstract

Since the discovery of high-temperature superconductivity, significant progress in the fabrication of REBCO-based (Rare Earth Barium Copper mixed Oxides) thin-films superconductors has been achieved. In our review, we described the approaches and possibilities of the improvement of superconducting properties by the introduction of nanosized pinning centers. We focused on the synthesis and viability of the material for artificial pinning centers and methods used for the introduction of the pinning centers into superconducting REBCO-based thin-films. This article summarizes available materials and procedures regardless of the financial cost of the individual method. According to available literature, the most significant superconducting REBCO tapes can be obtained when a combination of 1D and 0D nanoparticles are used for nanoscale pinning.

## 1. Introduction

Superconductors are a group of materials that have unique properties within the range of their critical parameters, such as zero electrical resistance and expulsion of the magnetic flux field from the superconductor’s volume [1]. The critical parameters are T_c_—critical temperature, B_c—_critical magnetic flux field, and J_c_—critical current density. Materials that exhibit a superconducting state at temperatures of 30 K (commonly) and above are known as high-temperature superconductors (HTS). Such materials were first discovered in 1986 by J.G. Bednorz and K.A. Miiller in the Ba-La-Cu-O system [2]. In practical use, it can be often seen that an HTS is referred to as a material with a critical temperature above 77 K as it allows employment of liquid nitrogen (LN2) as the cryogenic liquid in practical applications. Given that LN2 is readily available and relatively cheap in comparison with competing cryogenic solutions, most notably helium used for high field applications such as powerful magnets, as they allow for wider adoption of HTS [3,4,5,6,7,8].

Superconductors are divided into type I and type II. For type I superconductors, it is typical that they exhibit sharp and rapid change between the non-superconducting state and the superconducting state without any mixed region. In the case of type II superconductors, the transition from non-superconducting to superconducting is more complicated, and it is seen as more of a gradual process as they exhibit an intermediate mixed state [9,10,11,12,13]. The magnetic field penetrates into the type II superconductor in the form of magnetic vortices [14,15,16,17]. Such vortices tend to move in the superconductor in the direction given by Lorenz’s force which leads to a quenching of the superconducting state [18,19]. However, this behavior can to a significant extent be suppressed by pinning centers. As the vortices interact with pinning centers, this causes opposing force to the magnetic force generated by the supercurrent flowing in an infinite loop around the individual vortices. Simply put, pinning centers are non-superconducting regions, such as small impurities or crystal defects. Such pinning centers are crucial in the applications of superconductors as they can significantly influence critical parameters (most notably J_c_) [20,21,22]. Typical parameters for the most important superconducting materials, their forms, and usual critical parameters are summarized in Table 1.

REBa_2_Cu_3_O_7–δ_ or REBCO (rare earth element (RE)) is a group of type II ceramic high-temperature superconductors. REBCO superconductors are used, for example, in thin layers and films [28,29], second generation superconducting wires [30,31,32], or as the bi-functional catalyst for hydrogen evolution and oxygen reduction [33]. REBCO bulk ceramic is used in magnetic levitation objects for many applications [34,35], e.g., in precise positioners [36] or superconducting magnetic bearings [37].

Coated conductors with a thin film of REBCO are currently produced on an industrial scale by various companies worldwide, whilst the demand for these materials is steadily rising [38,39,40,41,42]. These thin-film coated REBCO superconductors are usually thin REBCO layers on very long (even several kilometers long) biaxially textured metallic tape with a thin intermediary multipurpose buffer layer of mixed oxides (usually) used to achieve proper crystalline orientation of the REBCO film and better adhesion on the metallic tape [43]. If the orientation (angle between individual grains) of REBCO thin layers varies too much, the supercurrent, hence J_c_ of the final thin film-coated superconductors are lowered by a large margin [44,45].

The phase composition of thin REBCO layers can differ significantly for different technologies. In general, the individual REBCO grains need to be properly aligned with very low relative angles at the grain boundaries in order to preserve the maximum critical current. Apart from that, the phase composition is mainly REBCO 123 (ReBa_2_Cu_3_O_x_), with a lower quantity of REBCO 211 (RE_2_BaCuO_5_). As the deposition of individual elements varies depending on the technique used, as well as on deposition parameters such as substrate temperature, quality of vacuum, acceleration voltage, etc. there can be a surplus of individual elements in the form of metal oxides or compounds which also belong to the individual REBCO system [46,47,48,49,50,51,52]. Additional phases, designed to serve as artificial pinning centers should be stable in the deposition conditions and chemically inert in relation to other present compounds in order to improve the properties of the final coated superconducting tapes [53]. There are several techniques for REBCO thin-film synthesis. The most common techniques used for the preparation of homogenous, high-quality superconducting films are summarized in Table 1, including name variants and abbreviations. The following compounds are generally used as substrates for REBCO films: SrTiO_3_, LaAlO_3_, MgO, yttria-stabilized zirconia, or sapphire with buffer layers of CeO_2_, Ag, MgO [54].

**Figure 1:**
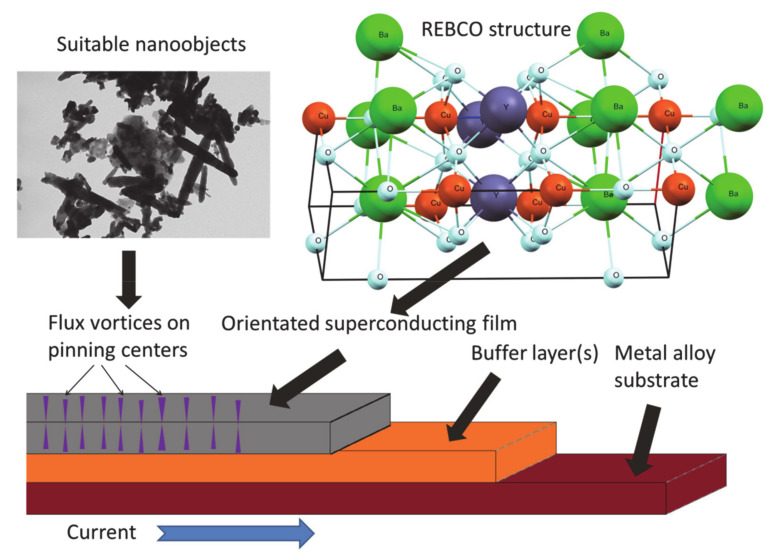
Scheme of a Rare Earth Barium Copper mixed Oxides (REBCO) thin-film tape with nanoparticles as a pinning centers.

The field of thin-film REBCO coated tapes has seen great progression over the last 40 years, with the optimization of a wide variety of factors—to name a few: texture and mechanical properties of metallic substrates, the stability of individual buffer layers and their influence on REBCO grain orientation and, finally, the properties of the final thin REBCO layer. Some of the deposition parameters, such as the heating of the sputtering targets, can yield significant improvements to the quality of the final tapes. These and many other factors studied in great detail in other papers [55,56,57,58] should also provide a consistent product while maximizing the operating hours and optimizing production costs. Balancing of these components is no mean feat, but as the worldwide production of thin-film, REBCO superconductors has matured and high-performance coated conductors are currently readily available worldwide. Coated superconductors of the second generation in general and REBCO coated superconductors particularly are especially useful for application in large and very large magnets in steady-state, where they are more economical alternative to standard resistive magnets, especially for maintenance and electricity costs and are the only sound alternative for generating large magnetic fields in large volumes [59]. Finally, the most significant advantage of coated REBCO superconducting tapes, in comparison with the other types of superconducting tapes, is the high value of critical stresses at cryogenic temperatures: over 800 MPa in the longitudinal tensile direction [60]. REBCO tapes can also easily be stacked on top of one another, forming twisted stacked cable conductors (TSTC), which are becoming more widespread in large powerful electromagnets and zero resistance superconducting cables in energy transport applications [61,62,63]. Typical REBCO thin-film tape with nanoparticles as pinning centers is visualized in Figure 1.

In this contribution, we would like to examine in detail the mechanism of artificial pinning in REBCO-based coated conductors, as well as the influence of various pinning methods, shapes of pinning centers, and approaches to the properties of thin-film REBCO coated conductors. The overview of the REBCO thin-film preparation methods is shown in Table 2.

## 2. Vortex Pinning in the Type II Superconductors

Type II superconductors have a transition region where the so-called mixed state appears. If a critical parameter is approached, the magnetic field enters into the mass of the superconductor in the form of so-called vortices. The vortices are surrounded by an infinite loop of supercurrents and therefore carry a magnetic momentum. The circular currents surrounding the vortex core make any vortices repel each other and forming a structured lattice, the so-called Abrikosov vortex lattice [10,73,74]. The vortices are subjected to Lorenz’s force if an electric current is passing through a superconductor. This means that if a depinning threshold is lower than Lorenz’s force, a flux line enters into the viscous-flow state and an electrical resistance can be observed as a result of the motion of such vortices [75]. This means that the transient state is always associated with the emergence of this movement in an ideal superconducting crystal. However, if there is a non-superconducting region in the superconductor, the vortex encounters such a defect, which is associated with the energetic minimum of the system, and the vortex will remain pinned to the defect center [76]. These regions of non-superconducting matter are therefore called pinning centers. In reality, an individual vortex usually interacts with a larger number of individual pinning centers, which provide an opposing force to the movement of vortices. The final movement is a superposition of individual force vectors that act upon the given vortex as a result of interaction with nearby pinning centers and the Lorenz force of the respective vortex itself [77,78,79,80].

Sizes, shapes, and the orientation of artificial pinning within the matrix are important for the pinning effect of the pinning centers. The dimensions of the pinning centers should ideally be in the order of the magnitude of the coherence length of a particular superconductor and for the cuprate superconductors, these will be nanoobjects. This translates to sizes from approximately 2–3 nanometers up to about 30–40 nanometers [81,82].

Artificial pinning centers can also be classified based on their dimensional structure. 1D artificial pinning centers are linear defects such as various dislocations or columnar defects. The columnar pinning centers can work better at higher temperatures, and their orientation in the matrix is one of the most important features since cuprate superconductors are highly anisotropic materials [82,83,84]. 2D artificial centers are defects located in planes, such as individual grain boundaries, line boundaries, stacking faults, or twin boundaries. 3D or bulk artificial centers such as voids, impurities, and pores can also serve as artificial pinning centers, but given that they are usually orders of a magnitude larger than the coherence length, their impact is fairly limited. These too can often lead to a decrease in mechanical properties, up to a point of failure, so they are often seen as more of a disadvantage. Special attention should be paid to point artificial pinning centers. These are often classified as a 0D artificial pinning centers, as they are often caused by nanoparticles [85,86]. Experiments show that the point defects have a maximum effect at temperatures below one half of the critical temperature; therefore, in superconductors working at the boiling point of nitrogen, their effect is limited [87], but they can still be of great importance, e.g., in high-performance magnets [88,89,90].

## 3. Methods of the Pinning Center Introduction into the Superconducting Thin-Films

There are several ways of introducing the pinning centers into the superconductors. Sufficiently small pinning centers of various types can be induced in the superconductor matrix through irradiation by various particles, e.g., by heavy ions [91], neutrons [92], or protons [93], or by electrons [94]. Irregular columnar pinning centers can be inducted in cuprate superconductors by addition of a small amount of ^235^U or ^209^Bi onto the superconducting matrix and subsequent bombardment of the material by thermal neutrons [95]. In general, there are several problems which irradiation poses, such as high cost, remaining radioactivity, and the fact that the pinning centers tend to disappear over time due to the thermal instability of the inducted defects [96].

Chemical pinning is another possibility of the introduction of the non-superconducting phase into superconductors. In this method, the reaction of various non-superconducting phases with the superconducting matrix itself results in local precipitates of a secondary phase or phases which are randomly oriented and dispersed in the superconducting matrix [97]. Unfortunately, secondary non-superconducting phases often have the potential for undesired shape and/or particle size. The fact remains that only a limited amount of these can be used and the increase in superconducting properties is not particularly intense anyway [97,98].

Furthermore, individual alien phases can react with the precursors, superconducting phase, and other additives. The advantage of this approach is its relative simplicity. Numerous compounds were tested as reacting additives, forming the pinning phases [99,100,101,102]. Metal oxides are frequently used for induction of chemical pinning centers, e.g., ZnO [103], TiO_2_ [104,105,106,107], or BiFeO_3_ [108]. While the introduction of chemical pinning centers has some advantages, such as lower complexity or being cheaper, in direct comparison with other methods, it lacks in the magnitude result. Therefore, for a better outcome, an addition of inert materials into a superconducting matrix is needed. The inert materials would keep its sizes and shapes, necessary to maximize the effect on the superconducting properties. Finding chemically inert substances which will persist during the procedure of the REBCO superconductor synthesis is not an easy task: especially given that such pinning centers are in the form of nanoobjects which tend to be more reactive due to their large surfaces, as can be seen, for example, in the case of nanosized ZnO [109]. Thermodynamic calculations can be very useful for identifying appropriate inert substances in comparison to extremely demanding experimental approaches [110,111], but such results need to be confirmed by experiments.

The introduction of nano-sized pinning centers into REBCO films has dramatically improved the J_c_–B characteristics of films at various temperatures and fields. The method of pinning center introduction is dependent on the method of thin-film preparation. In the case of PLD, the pinning centers or their precursors are usually present in the original target in a suitable form, e.g., yttria-stabilized zirconia [112], BaZrO_3_, or Y_2_BaCuO_5_ [113], and pinning structures are formed during film deposition and processing. Using the sputtering-based methods, pinning centers can be deposited in the forms of islands on the substrate before the final deposition of the REBCO film. Pd or Ta nano-islands were deposited by dc-magnetron sputtering on LMO/homo-epitaxial MgO/IBAD-MgO/Hastelloy templates in argon and argon and hydrogen atmospheres. Subsequently, YBCO film was deposited in the usual oxidative atmosphere by PLD, resulting in increased pinning [114]. The opposite approach is also possible. A Zn precursor was deposited on a substrate by MOCVD and ZnO nanorods were synthesized by thermal treatment. Subsequently, a YBCO film was created on the ZnO nanorods by DC magnetron sputtering deposition [115].

In the case of methods based on the direct deposition of liquid precursors, the pinning centers or their precursors can be part of the original raw mixture. Zr-naphthenates was added into the precursor solution of tri-fluoroacetate salts to introduce BaZrO_3_ flux pinning centers into the YBCO films prepared by the MOD method [116]. A similar approach was used with Sn-doped YBCO film, also prepared by MOD, resulting in the presence of a YBa_2_SnO_5.5_ pinning phase [117]. Furthermore, in the case of indirect transport methods as MOCVD, the precursors for pinning centers are usually added into the raw material and transported, with the material, to the film. The resulting pinning centers are subsequently formed by chemical reaction, as in the case of Y(thd)3, Ba(thd)2*tetraglyme, Cu(thd)2, Zr(thd)4 as precursors in the form of the mixture of the precursor powders. The resulting YBCO film contained BaZrO_3_ nano-inclusions [118]. The problem with the introduction of the pinning material in the form of the precursor can be that it is difficult to obtain phase-oriented dispersed pinning as needed. However, for example, BaMO_3_ nanorod growth in REBCO superconducting thin films prepared by the vapor phase epitaxy can be simulated by the three-dimensional Monte Carlo method [119].

## 4. Artificial Pinning Centers in RECBO Thin-Film Superconductors

The evaluation of various pinning centers can prove to be a significant challenge insofar as mere synthesis of consistent, well-oriented REBCO coated superconductors have taken decades of dedicated research. While advances in computer modelling can yield interesting results, the complexity of the behavior of vortices can prove to be very challenging in the face of designing REBCO coated superconductors with effective artificial pinning. Sorting out the contribution of various pinning centers, given the temperature, types of pinning, and naturally occurring defects is experimentally challenging [16,120,121,122]. Local isotropy and anisotropy caused by the pinning center itself can also play a significant role and can be evaluated by rotating an applied magnetic field while measuring J_c_, which allows for separation of isotropic effects, as they are indifferent to the field orientation, according to Blatter scaling [123,124,125].

Artificial pinning with the usage of noble metals, such as silver [126,127,128,129], gold [130,131,132], palladium [133], or platinum [134,135], is known to be possible, with the major disadvantage being that noble metals are quite expensive. The usage of inert ceramics from the same REBCO system is also a possibility, for instance in the case of YBCO system, phase Y-2411-M (Y_2_Ba_4_CuMO_x_), where M = Nb, Ta, Mo, W, Ru, Zr, Bi and Ag phase seems to be a promising material [136,137,138,139,140,141,142]. Similarly, inert perovskite phases (stable at high temperatures) such as BaZrO_3_, Ba_2_RETaO_6_, Ba_2_Y (Nb, Ta)O_6_ or BaTiO_3_ have been also reported as prospective candidates for inert pinning centers [143,144,145,146]. An overview of achieved results with various phases used as artificial pinning centers can be seen in Table 3.

**Figure 2:**
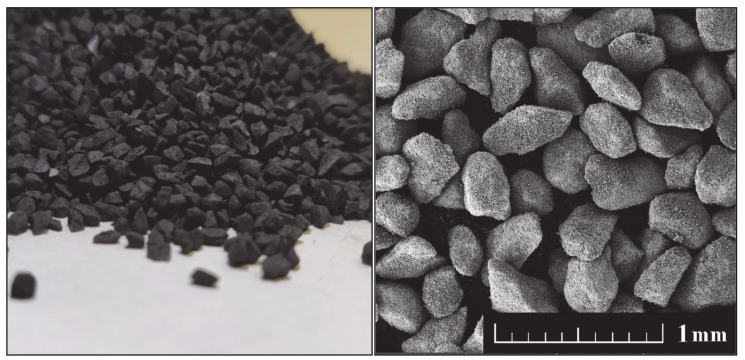
An example of a commercial granulate used for the deposition of thin-film superconducting tapes; photography of GdBCO granulate (**left**), SEM micrograph of GdBCO granulate (**right**).

The design and development of superconducting REBCO-based coated wires is highly dependent on the method used. Techniques such as CVD or PVD are based on simultaneous growth, hence the orientation of individual phases tends to be determined by the growth mechanism, resulting in the overall defined orientation of artificial pinning centers [40,152,153]. The results can differ significantly for deposition techniques which result in the partially random orientation of artificial pinning centers such as various sputtering techniques. It has been shown that while the artificial pinning centers retain a part of orientation on the surface of the thin layer, these particles are randomly oriented in the bulk of the layer. This means that while the orientation of the pinning centers in granulates does not matter since the individual grains are randomly oriented (see Figure 2), this adds further requirements to the already complicated production of sputtering targets. The effect of such techniques can differ in respective directions, given that 1D artificial pinning centers, such as columnar defects or nanorods, have shown significant improvements in J_c_. To combat this issue, a combination of different artificial pinning centers has been developed. A synergy between 1D and 0D pinning centers had been shown for various perovskite-based phases (forming 1D nanorods) with nanoparticles at the same time [121,154,155]. A well-known combination of these artificial pinning centers is BaZrO_3_ and Y_2_O_3_ [156,157,158] (similar behavior had also been reported in similar perovskites such as BaSnO_3_ [159] and BaHfO_3_ [160]). Nanomaterials have specific properties that should be taken into consideration before use. Their large surfaces cause a considerable change in their physical and chemical properties, which need to be taken into account while designing coated REBCO tapes, as this can dramatically alter their interaction with the REBCO matrix and result in undesired behavior.

**Figure 3:**
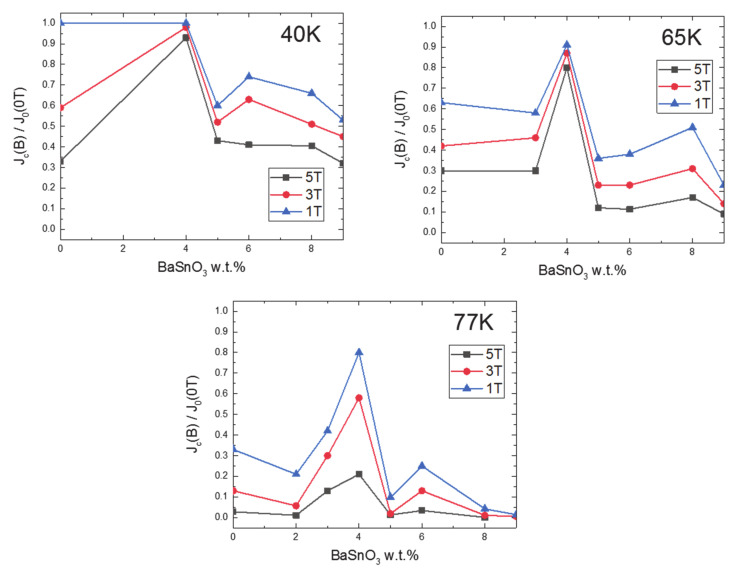
Normalized critical current densities of BaSnO_3_-doped YBCO films for various BaSnO_3_ content, obtained at 1 T, 3 T, and 5 T at 40 K, 65 K, and 77 K. The results show that the optimal content of BaSnO_3_ is approx. 4 wt. % [167].

Although the vast majority of published articles deal with pinning properties at the temperature of boiling nitrogen, pinning has the biggest impact at low temperatures (4 K), which are achievable by using liquid helium. Nevertheless, an increase of the pinning effect is noticeable even for higher temperatures [149,161] achievable more economically by various types of cryo-coolers [162,163,164,165]. This is important for applications in superconducting magnetic energy storage devices, motors, and generators which usually work in the range of 30–65 K and a mid-to-high magnetic field. On the other hand, high field magnets used, for instance, in nuclear fusion and sophisticated high energy physics applications need helium temperatures to work for the production of very high magnetic fields. [166] In general, the methods of the introduction of pinning centers are generally the same for all applications. An example of the temperature dependence of the critical current density depending on the pinning and temperature can be seen in Figure 3, where normalized critical current densities of BaSnO_3_-doped YBCO films are shown for different BaSnO_3_ concentrations, at different temperatures in various fields [167].

In particular, the low temperature-high magnetic field operational interval is so important for high energy physics and nuclear fusion applications that it has drawn ever-increasing interest in recent years from both the scientific community and industry. High-field hybrid magnets, which use a very long YBCO tape can achieve impressive magnetic fields of up to 45 T [168].

## 5. Synthesis and Viability of Material for Artificial Pinning Centers for the REBCO Coated Superconductors

**Figure 4:**
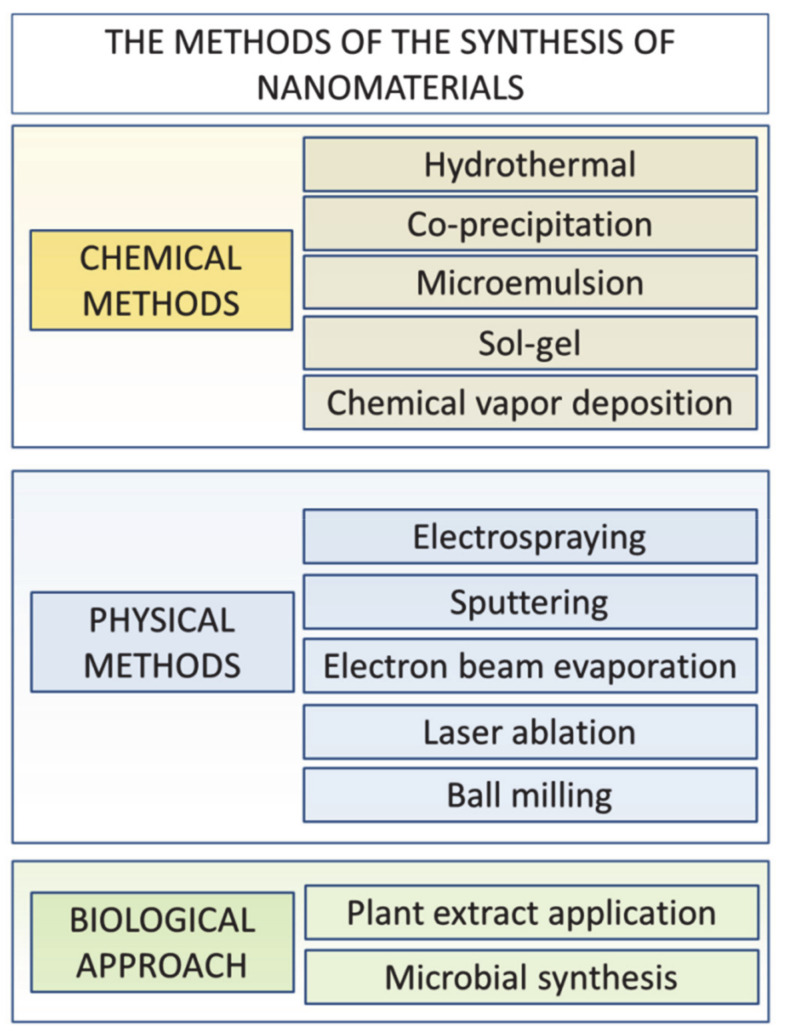
The overview of possible methods and approaches to nanomaterial synthesis.

The addition of chemically inert material in the form of nano-objects of desired shapes by the optimized process which enables its preferred orientation is certainly one of the ways of introducing working pinning centers into the YBCO films. Purchasing the nanoparticles from commercial vendors is usually the first choice of any group dealing with research or commercial needs for REBCO thin-film synthesis. There are numerous commercial subjects that can supply a sufficiently large amount of broad spectrum of the desired nanomaterials. However, this approach has its limits. Some suitable materials are not available or are not available in sufficient quality or amount. Further, price and time of delivery may play a role. In any event, knowledge of the exact synthetic route and thoughtful characterization of the materials may be crucial in any material research. As a result, the synthesis of one’s own nanomaterials may be important. Few requirements are crucial for choosing synthetic procedures. To name a few: quality, particle size, morphology, purity of synthesized material and sufficient amount of prepared material. The chemical synthesis of nanomaterials by sol-gel processes is an effective method for producing high-quality metal oxide nanoparticles [169]. The sol-gel methods usually yield a sufficient amount of material but it may sometimes be difficult to obtain very fine nanoparticles or the desired morphologies. Wet chemical synthesis, such as solvothermal and hydrothermal synthesis, as well as microwave-assisted or flow synthesis, may also be possible methods from which to choose [170]. This family of synthesis offers wide possibilities in terms of control of sizes and shapes of oxide metal nanomaterials. Further, the thermal decomposition of powder precursors may produce relatively large amounts of mixed oxide nanomaterials [171,172,173]. Even biosynthesis of the various metal-oxide nanoparticles—for example, bacteria-mediated actinomycetes biogenic synthesis—is possible [174]. Nevertheless, this kind of synthesis seems to be more suitable for the preparation of stable colloid systems than large quantities of powder material. An overview of possible synthetic methods can be found in Figure 4.

**Figure 5:**
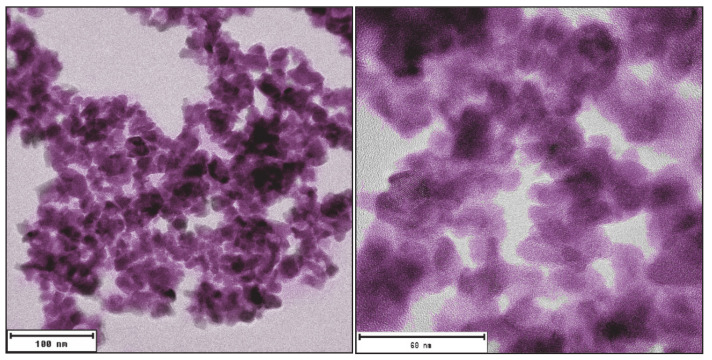
Colorized TEM micrographs of fine BaZrO_3_ nanoparticles with an average particle size of approximately 10 nm synthesized by the wet-chemical method.

More specifically, the perovskites of the BaMO_3_ type, e.g., BaZrO_3_, can be prepared in following ways. Fine BaZrO_3_, BaTiO_3,_ and LiNbO_3_ nanoparticles were synthesized in gram quantities by a soft chemistry route. The typical microstructure of fine BaZrO3 is shown in Figure 5. The preparation is based on the dissolution of alkali or alkaline earth metals in benzyl alcohol and a subsequent reaction with transition metal alkoxides at relatively low temperatures of 200–220 °C. All the as-synthesized particles are highly crystalline and are tiny [175]. Another method of preparation of the BaZrO_3_ nanoparticles is based on solvothermal and microwave-assisted synthesis [176]. BaZrO_3_ nanoparticles from approx. 40 nm further can be synthesized through the calcination of precursors prepared by the auto-combustion sol-gel method based on the citrate-nitrate approach [167]. These methods can be adapted for the synthesis of the large amounts of the material needed for the experiments with nano-pinning in REBCO. In contrast, the application of some methods such as synthesis by reverse micelle approach [177] may be more challenging due to the difficulty of obtaining large quantities of synthesized BaZrO_3_ nanoparticles.

## 6. Conclusions

In conclusion, REBCO coated superconducting tapes technology has matured significantly over the last decades, both in terms of the technology itself and the global production of high-performance tapes. This advancement has allowed for steady growth in application areas that rely heavily on second generation coated superconducting tapes, most notably high-field hybrid magnets, which use miles of REBCO tapes in order to achieve fields of up to 45 T, with the potential for wide applications in other fields such as energy transport, research, and many more. In this review, we have focused on the mechanism, engineering, and results of artificial pinning centers in the field of high-performance thin-film coated superconducting tapes. While the general idea for artificial pinning is a simple one, the successful implementation of the theory behind the behavior of vortices and pinning centers still proves to be challenging. It is apparent that pinning centers of various sizes, morphology, and chemical compositions need to be employed, depending on the deposition techniques and intended use of the final product. The improvement for various pinning phases has been arranged into a table for a clear comparison. It seems that for superconducting REBCO tapes, the best overall approach is a combination of 1D (columnar defects and nanorods) and 0D (nanoparticles). This combination provides high-performance tapes whilst allowing for a slightly larger margin of error, given that the orientation of the 1D pinning centers is just one contribution of total pinning present in the REBCO layer. Finally, the methods of producing artificial pinning centers—most importantly, nanoparticles and nanorods—were discussed.

## Figures and Tables

**Table 1:** Overview of the forms and usual critical parameters of typical superconductors in the conditions they are commonly used. Let us note that the J_c_ values are without any enhancements such as artificial pinning centers, to which is devoted Table 3.

Superconductor	Typical Forms/Application	Usual Critical Parameters
Low-temperature Nb_3_Sn superconductor	Composite wire-magnets	T_c_ = 18.3 K, upper critical magnetic field (H_c2_) up to 35 T, [23]
MgB_2_	Silver/steel sheeted wires-magnets, current lead	T_c_ = 39 K, 10^9^ A/m^2^ at 5 T, H_c2_ up to 74 T in thin films, [24]
YBaCuO	Tapes, films, bulk-magnets, levitation bulks, current lead	123 phase: T_c_ = 92 K, J_c_ 2.8 MA/cm^2^ self-field, 77K, [25]
Bi(Pb)SrCaCuO	Tapes, bulk-current lead, levitation bulks, magnetic screens	2223 phase: T_c_ = 108, J_c_ = 5 kA/mm^2^ self-field,77 K, [26]
REFeAsO	Experimental samples	T_c_ = 55 K for SmFeAsO–metallic behavior form T_c_ to 300 K, [27]

**Table 2:** Overview of the REBCO thin-films preparation methods.

Method (Abbreviation)	Method Principle	Ref.
Pulsed laser ablation (PLA)/Pulsed laser deposition (PLD)	Film deposition by PLD by irradiation of a target by a laser beam. The laser beam removes material from the target and material is moved to the substrate.	[64,65]
Metalorganic chemical vapor deposition (MOCVD)/Metalorganic vapor-phase epitaxy (MOVPE)	Organometallic precursors are injected together with carrier gasses and thin-film grows due to chemical reactions on the substrate	[66,67,68]
Chemical solution deposition (CSD)/Metal-organic deposition (MOD)	Deposition of chemical precursors, usually trifluoroacetate (TFA; MOD-TFA method) on the substrate, e.g., By dip-coating, followed by pyrolysis, heating, film growth and oxidation processes	[69,70]
Ion plasma sputtering (no abbreviation common)	Argon and oxygen plasma, in the magnetic field, is used for sputtering of target form REBCO material onto the layer	[71,72]

**Table 3:** Examples of the pinning materials and results in selected REBCO 2D superconductors.

Pinning Material	Type of Superconducting Material	Improvement in J_c_ (if Applicable) or Maximal J_c_ at the Respective Conditions	Reference
BaZrO_3_	YBCO film	5 J_c_-self field	[143]
Ba_2_RETaO_6_	YBCO film	6 J_c_-self field	[144]
Ba_2_Y(Nb,Ta)O_6_	YBCO film	10 MA cm^−2^, 30 K, 5 T	[145]
BaTiO_3_	YBCO film	8.42 MA cm^−2^-self field	[146]
BaZrO_3_ and Y_2_O_3_	YBCO delta film	2.3 MA cm^−2^ (6.4 µm thickness)	[147]
BaZrO_3_	YBCO delta microtapes	J_c_ at 1 T of 3 MA cm^−2^	[148]
BaHfO_3_	GdBCO thin film	6 J_c_-self field, 10 K, 6T	[149]
BaHfO_3_	YBCO delta film	3.5 J_c_ 77 K, 1T	[150]
Y_2_O_3_	YBCO film	2.25 J_c_, 77 K, 5T	[151]
Ag	YBCO delta film	2.5∙J_c_ self field	[129]
Au	YBCO delta film	1.7 J_c_ self field	[132]
